# Neuromodulators and Long-Term Synaptic Plasticity in Learning and Memory: A Steered-Glutamatergic Perspective

**DOI:** 10.3390/brainsci9110300

**Published:** 2019-10-31

**Authors:** Amjad H. Bazzari, H. Rheinallt Parri

**Affiliations:** School of Life and Health Sciences, Aston University, Birmingham B4 7ET, UK; parrihr@aston.ac.uk

**Keywords:** neuromodulators, synaptic plasticity, learning, memory, LTP, LTD, GPCR, astrocytes

## Abstract

The molecular pathways underlying the induction and maintenance of long-term synaptic plasticity have been extensively investigated revealing various mechanisms by which neurons control their synaptic strength. The dynamic nature of neuronal connections combined with plasticity-mediated long-lasting structural and functional alterations provide valuable insights into neuronal encoding processes as molecular substrates of not only learning and memory but potentially other sensory, motor and behavioural functions that reflect previous experience. However, one key element receiving little attention in the study of synaptic plasticity is the role of neuromodulators, which are known to orchestrate neuronal activity on brain-wide, network and synaptic scales. We aim to review current evidence on the mechanisms by which certain modulators, namely dopamine, acetylcholine, noradrenaline and serotonin, control synaptic plasticity induction through corresponding metabotropic receptors in a pathway-specific manner. Lastly, we propose that neuromodulators control plasticity outcomes through steering glutamatergic transmission, thereby gating its induction and maintenance.

## 1. Introduction

A huge emphasis has been put into discovering the molecular pathways that govern synaptic plasticity induction since it was first discovered [1], which markedly improved our understanding of the functional aspects of plasticity while introducing a surprisingly tremendous complexity due to numerous mechanisms involved despite sharing common “glutamatergic” mediators [2]. The considerable variation in the signalling pathways and induction requirements, mostly attributable to various methodological approaches across heterogeneous neuronal populations, itself is key to comprehend and properly stratify the diverse mechanisms by which specific neuronal subclasses control their synaptic strength and structure in association with learning, memory, sensory and motor functions [3,4]. However, at least in relation to long-term synaptic plasticity, the focus has been directed predominantly towards glutamatergic transmission, particularly the roles of *N*-methyl d-aspartate receptors (NMDARs) and calcium/calmodulin-dependent protein kinase II (CAMKII). Nonetheless, an extensive number of studies show critical roles for other neurotransmitters and modulators in long-term potentiation (LTP) and depression (LTD) through corresponding G-protein coupled receptors (GPCRs). In this review, we explore current evidence on the roles of neuromodulators as key effectors in the induction and maintenance of long-term synaptic plasticity and associated GPCRs involvement in the modulation and steering of glutamatergic transmission focusing on plasticity induction, which could facilitate future development of models determining the mechanisms that underlie certain cognitive, behavioural, learning and memory subtypes.

## 2. Metabotropic Pathways Are Necessary for Long-Term Synaptic Plasticity

Long-term synaptic plasticity (i.e., LTP and LTD) can be generally classified either based on the loci of expression into presynaptic and postsynaptic forms or based on the molecular mediators into two broad categories, NMDAR-dependent and NMDAR-independent forms, both of which have been reported in different brain regions. It is thought that the “early” phase of LTP (e-LTP) mediates short-term “labile” memories that still require maintenance and the late phase for long-term storage [5,6,7]. Despite previous evidence in relation to NMDAR-dependent LTP showing key roles for CAMKII not only in the induction but also the maintenance of LTP [8,9,10], other studies show the opposite and it is proposed that the use of structural CAMKII inhibitors is difficult to interpret as they might affect baseline transmission [11,12,13,14]. However, it is well-known that long-term storage of plasticity-induced memories requires in the vast majority of cases gene expression, which is often referred to as the protein-synthesis phase of plasticity that is necessary for long but not short-term memory formation [15,16,17,18,19]. Accordingly—at least at specific synapses—the activation of certain GPCRs during plasticity induction is found to trigger the gene-expression and/or translation of messenger ribonucleic acid (mRNA) of proteins implicated, either functionally or structurally, in plasticity regulation, maintenance and memory consolidation such as brain-derived neurotrophic factor (BDNF), activity-regulated cytoskeleton-associated protein (Arc), fragile-x mental retardation protein (FMRP) and others [20,21,22]. 

It was previously shown that NMDAR-dependent LTP involves local protein synthesis, which precedes gene expression, or de novo protein production; however, various studies report that it is induced via activation of BDNF, dopamine and metabotropic glutamate receptors (mGluRs) [23]. In addition, local protein synthesis is also observed in NMDAR-independent forms of both LTP and LTD; for instance, through BDNF signalling and mGluRs, respectively [24,25]. Therefore, NMDAR-mediated activation of CAMKII auto-phosphorylation does not fully and solely explain both LTP induction and maintenance. However, recent evidence shows ion flux-independent metabotropic roles of NMDARs particularly in LTD, as some studies report that use-dependent blockade of NMDAR ion channel using dizocilpine (MK-801) is found to block LTP but not LTD in contrast to competitive antagonism using D-AP5 which blocks both [26]. With regards to NMDAR-independent plasticity in which GPCRs, most commonly mGluRs, have primary functions it might be argued that one exception is the voltage-gated calcium channel (VGCC)-dependent long-term plasticity in which VGCCs can induce NMDAR-independent plasticity and may be sufficient for induction [27,28,29]. However, VGCC-dependent LTP and LTD are also found to depend on either mGluRs (e.g., hippocampus and cerebellum) or both mGlu and NMDA receptors (insular cortex) [30,31,32]. Despite that different induction protocols, tissue preparations and experimental conditions can lead to varying results that should not be directly compared, experimental findings show that some plasticity mechanisms are shared by various neuronal populations and that the same synaptic population can exhibit different mechanisms for plasticity induction—with distinct expression mediators—that could have independent functional outcomes. 

Another mechanism by which GPCRs mediate both early and late phases of plasticity is through the activation of protein kinases, which can mediate the expression, trafficking and synaptic insertion of NMDA and/or AMPA (α-amino-3-hydroxy-5-methyl-4-isoxazolepropionic acid) receptors through downstream signalling, such as protein kinase A (PKA), PKC, PKMzeta, mammalian target of rapamycin (mTOR), phosphoinositide 3-kinase (PI3K) and mitogen-activated protein kinases (MAPK) all of which have been shown to be involved, either differentially or synergistically, in plasticity induction and/or maintenance [33,34,35,36,37]. In addition, with regards to the critical roles of astrocytes, GPCR activation and subsequent induction of astrocytic calcium signalling is found to trigger gliotransmitter release that can subsequently induce multiple forms of long-term plasticity either directly through both pre- and postsynaptic mechanisms or indirectly via altering neurotransmission to augment or prevent plasticity induction [38,39]. Another possible mechanism for GPCRs in synaptic plasticity is based on intracellular calcium mobilisation during induction as seen with mGluRs in cerebellar LTD [40]. Lastly, evidence shows G-protein-specific effects in plasticity outcome modulation; for instance, the activation of hippocampal (CA1) mGluR5 lowers the threshold for (i.e., promotes) LTD, while group II mGluRs prime NMDAR activation and facilitate LTP. On the other hand, mGluR3 blockade in the dentate gyrus impairs post-synaptic LTD while its activation modulates LTD presynaptically and impairs LTP; similarly, blocking group III mGluRs impairs LTD and long-term spatial memory but not LTP in CA1 neurons [41,42,43,44]. Additionally, mGluR activation is found to control the polarity of plasticity; for instance, in acute spinal slices of substantia gelatinosa, blocking mGluRs switches spike timing-dependent plasticity (STDP) following a pairing stimulation protocol from LTP to LTD, while, in hippocampal slices, the activation of mGluRs switches chemical LTD (cLTD) induced by brief application of NMDA into sustained LTP [45,46].

## 3. Neuromodulators in Synchronisation and Synaptic Plasticity

Accumulating evidence illustrates critical plasticity roles for GPCRs, particularly mGluRs, through differential signalling pathways, and shows the involvement of other transmitters and modulators as key participants in synaptic plasticity and memory. It is evidently supported that long-term synaptic plasticity is the molecular mechanism of learning and memory as shown by evidence addressing the assessment criteria of the synaptic plasticity and memory (SPM) hypothesis [47]. Furthermore, since at its core long-term plasticity is a neuronal encoding mechanism, it is plausible to assume its involvement in the control of behaviour which is induced by previous experience. Indeed, evidence shows a strong correlation between synaptic plasticity and experience-dependent (i.e., motor and sensory) learning [48,49]. In addition, synaptic plasticity is found to be physiologically involved in stress, fear and emotional memory as well as pathologically in anxiety, major depressive disorder (MDD) and drug addiction [50,51,52,53,54,55]. It is well-known that the majority of aforementioned psychological, behavioural and learning subtypes rely heavily or at least partly on cholinergic, dopaminergic, serotonergic or adrenergic transmission [56]. 

The artificial—mostly in vitro—induction of long-term plasticity in acute slices or primary cultures requires the use of certain stimulation protocols suggested to mimic physiological patterns of network activity required for synaptic plasticity and memory, most commonly high-frequency, theta-burst and low-frequency stimulation (HFS, TBS and LFS, respectively) as well as pairing protocols [57]. It is strongly believed that brain rhythms represent oscillatory network activities responsible for multiple emotional, behavioural and memory functions. In addition, it is proposed that the clinical consequences of transcranial magnetic stimulation at certain frequencies are mediated by either short or long-lasting synaptic plasticity mechanisms [58,59]. Indeed, clinical and in vivo evidence shows frequency and network-based correlations between single-band/coupled brain oscillations and memory retention and/or retrieval; for instance, theta, theta/gamma, beta/gamma, theta/theta synchronous and alpha/gamma rhythms in different brain regions associate with spatial, associative, olfactory, fear and visual memories, respectively [60,61,62,63,64]. 

Neuromodulators provide fine regulation of network activities and are suggested to control frequency-dependent transmission [65]. Accordingly, evidence indicates that neuromodulators couple rhythmic activity and synaptic plasticity; for instance, hippocampal muscarinic M_1_ receptors are critical for theta-oscillations, LTP induction and spatial memory performance [66]. Moreover, increased expression of serotonin reuptake transporter blocks theta-oscillations in the amygdala and impairs fear learning while its deficiency causes delayed extinction learning and increased amygdala–prefrontal cortex (PFC) theta-synchronisation [67,68]. Furthermore, noradrenaline release following direct stimulation of locus coeruleus is reported to induce narrow-theta (7–9 Hz) oscillations and LTP in the dentate gyrus while in hippocampal CA1 region it results in transient theta suppression and LTD facilitation through β adrenergic receptors [69,70]. Additionally, selective activation of dopamine D_4_ receptor—which is associated with attention and working memory—induces gamma oscillations in the hippocampus though parvalbumin-positive interneurons; in contrast, D_3_ activation inhibits gamma oscillations and certain memory functions, whereas blocking D_1/5_ receptors during memory-encoding impairs long- but not short-term retention [71,72,73]. On the other hand, low-frequency (slow-wave) delta oscillations are mostly observed during non-rapid eye movement (NREM) sleep. Slow-wave oscillations are hypothesised to downscale synaptic strength of synapses potentiated during wakefulness that would otherwise become saturated [74]. Little is known about the roles of modulators in slow-wave oscillations; however, the oscillations are found to be partly regulated by astrocytic adenosine-mediated inhibition through A_1_ receptors; additionally, blocking activity-driven BDNF expression reduces both NREM slow-wave oscillations and synchronous PFC–hippocampal theta oscillations during extinction learning [75,76]. Lastly, GPCR-induced expression of certain immediate early genes, namely c-Fos and fos B genes, is found to regulate slow wave sleep and delta oscillations [77].

**Figure 1:**
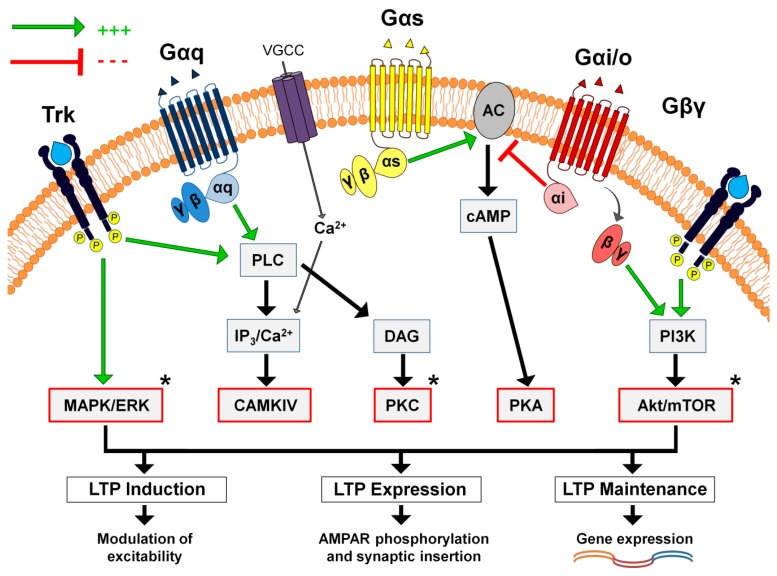
Schematic representation of the main metabotropic signalling pathways by which neuromodulators control long-term potentiation (LTP). Neuromodulators activate various protein kinases that can: Firstly, modulate neuronal excitability through controlling ion-channel conductance (e.g., NMDAR and VGCC) leading to the facilitation or direct induction of LTP. Secondly, protein kinases (e.g., PKA, MAPK and PKC) can initiate the expression, trafficking, phosphorylation and/or synaptic insertion of AMPA receptors leading to LTP expression. Lastly, protein kinases trigger the gene expression of proteins necessary for LTP maintenance. * It is also implicated in long-term depression (LTD), mostly through AMPA receptor internalisation.

The above findings provide direct evidence for the essential involvement of neuromodulators in both brain oscillations and memory processes. Despite extensive efforts to unravel the association between rhythmic brain activity and various behavioural and cognitive functions, the specific roles of neuromodulators remain incompletely understood. Additionally, network activity regulation is most likely resultant from balanced effects of multiple transmitters and modulators that vary between different brain states. Lastly, the metabotropic pathways by which neuromodulators control long-term synaptic plasticity, summarised in Figure 1 below, are shared by various neuromodulators, hence suggesting activity/behaviour-dependent and pathway-specific mechanisms. Therefore, the roles of neuromodulators in synaptic plasticity need to be investigated in relation to specific innervation pathways as well as associated learning subtypes through characterising their direct (i.e., synaptic) involvement in glutamatergic steering and plasticity induction, as discussed below.

## 4. Transmission System-Based GPCR Modulation of Plasticity

### 4.1. Dopaminergic Transmission

Dopamine is the most-extensively studied neuromodulator in relation to synaptic plasticity due to its crucial roles in emotion and motor control in addition to its non-fully characterised but still critical involvement in cognition [78]. Despite subtype-specific polymorphisms, dopamine receptors are classified into two main classes, namely D1 and D2 receptors, also known as D_1_-like and D_2_-like receptors (subscript denotes receptor subtypes), respectively, which are widely but differentially expressed across the brain. The former (D1) comprises D_1_ and D_5_ receptor subtypes while the latter (D2) comprises D_2–4_ subtypes [79]. D1 receptors are coupled to Gs-protein, the activation of which stimulates adenylyl cyclase and subsequent cAMP (cyclic-adenosine monophosphate) production and PKA activation, while D2 receptors are coupled to Gi-protein and produce the opposite effect leading to reduced cAMP production. However, dopamine receptors have been shown to stimulate or inhibit other signalling pathways and intracellular effectors including Ca^2+^ channels, K^+^ channels, Na^+^/H^+^ exchanger and Na^+^/K^+^ ATPase pump in addition to critical roles in the induction and regulation of gene expression [80]. 

Dopaminergic pathways have been identified for decades; however, the clinical aspects, newly-identified inputs and projections and the concept of co-transmission represent more recent insights into dopamine signalling [81,82]. Midbrain dopaminergic neurons in the ventral tegmental area (VTA) and substantia nigra pars compacta (SNc) represent the two predominant sources of brain dopamine as a modulator of neurotransmission, that project to distinct brain regions [83]. More recent evidence shows that VTA and SNc exhibit differential connectivity covering the majority of brain regions. In addition, direct electrical and optogenetic stimulation of VTA dopamine release causes brain-wide network activity modulation even in regions receiving minimal to no VTA innervation [84,85]. However, in relation to long-term synaptic plasticity and associated learning and memory, the greatest body of evidence exists for mesocortical, nigrostriatal and mesolimbic pathways.

#### 4.1.1. Mesocortical Pathway

Mesocortical projections from the VTA extend to the PFC and other cortical regions, particularly the primary motor cortex (M1-cortex). The roles of PFC dopamine in learning and memory are well-documented in relation to context encoding, spatial working memory, and emotional and associative learning mostly through phasic release [86]. Functional coupling between the PFC and VTA is shown to be mediated through phasic bidirectional control over sub-second timescales, which is found in computational models to be important for mediating and determining the maintenance and magnitude of long-term plasticity, respectively [87,88]. In addition, dopaminergic control of PFC activity is suggested to regulate the thresholds for LTP and LTD [89]. 

Evidence shows critical roles for dopamine in LTP and LTD of PFC neurons as D_1_ receptor is found essential for the maintenance but not induction of LTP while its agonist activation facilitates LTD induction through transforming transient LFS-induced depression into sustained LTD [90]. Additionally, dopamine is also reported to be essential for NMDAR-dependent LTP induction in vivo at hippocampal–PFC synapses through a D1- and PKA-dependent mechanism [91]. Moreover, direct VTA stimulation shows frequency-dependent modulation of plasticity and synaptic transmission at hippocampal–PFC synapses, highlighting the importance of mesocortical dopaminergic transmission in the induction of PFC long-term plasticity [92]. In vitro evidence shows that dopamine can facilitate either LTP or LTD at PFC Layers I/II–V synapses, in which brief dopamine application induces LTD and facilitates tetanic stimulation-induced LTD as well. On the other hand, when dopamine application precedes tetanic stimulation, it facilitates NMDAR-dependent LTP, suggesting an activity-dependent role of “background” dopamine, which reflects concurrent synaptic activation and NMDAR priming, in determining the polarity of plasticity [93,94]. Additional evidence for the critical involvement of dopaminergic transmission in long-term plasticity comes from novel forms of associativity; as dopamine-facilitated LTD following tetanisation is found to depend on simultaneous mGluR activation and synergistic activation of MAPK, cooperative activation of D1 and D2 receptors additionally allows extended timing-dependent LTP (t-LTP) through neuronal excitation and inhibition of GABAergic transmission, respectively [95,96]. 

Most established evidence for PFC dopamine shows key roles in working memory and consolidation especially at hippocampal–PFC synapses as opposed to mostly-glutamatergic induction of long-term plasticity [97]. However, recent studies unveiled various mechanisms by which dopamine receptors modulate glutamatergic transmission to augment or depress PFC plasticity induction; for instance, D2 receptor activation depresses NMDAR transmission and disrupts synaptic function at hippocampal–PFC synapses, whereas at Layer V neurons D1 activation strengthens and depresses NMDAR-mediated and non-NMDAR-mediated excitatory currents, respectively, while decreasing neurotransmitter release following moderate-frequency (20 Hz) stimulation [98,99]. Moreover, dopamine receptors can directly affect NMDA and AMPA receptor expression, as D1 activation induces NR1/NR2B-containing NMDAR expression and trafficking in PFC neuronal cultures through a tyrosine kinase-mediated PKA-independent mechanism; moreover, D1 activation is found to induce PKA-dependent expression of surface GluA1 (formerly known as GluR1) subunit-containing AMPARs, which is suggested to be an LTP-facilitating mechanism, while D2 activation decreases synaptic expression of AMPARs in primary PFC cultures [100,101]. Interestingly, D1 receptors co-localise with NMDARs in PFC pyramidal neurons and D1 activation is found to potentiate NMDAR-mediated calcium influx through a PKA-mediated mechanism [102]. Furthermore, the activation of D_4_ receptor subtype on PFC GABAergic interneurons supresses their glutamatergic transmission through regulating calcineurin-dependent AMPAR trafficking; accordingly, D_4_-selective activation results in decreased neuronal and interneuronal (following a transient increase) spontaneous action potential frequency in contrast to dopamine itself, which depresses and increases neuronal and interneuronal firing, respectively [103,104]. 

The critical involvement of dopamine in various functions of the PFC including executive function, attention and learning are indirectly supported by pathology and drug-induced effects on PFC plasticity; for instance, D2 receptor overactivation is found to induce LTD and subsequent long-lasting suppression of NMDAR transmission of hippocampal–PFC synapses as a suggested mechanism of schizophrenia-associated cognitive deficits [98]. On the other hand, low-dose amphetamine induces cAMP-PKA mediated LTP through D1, but not D2, and adrenergic receptors. However, higher doses impair potentiation through both D1 and D2 receptors by activating protein-phosphatase 1 (PP1), similar to the observed effects of inhibited dopamine reuptake, which impaired D1/D2-dependent MAPK (ERK1/2)-signalling-mediated LTD through D1 overactivation that was rescued by positive allosteric-modulation of mGluR5 [105,106]. Lastly, D1-induced PKA/mTOR signalling-dependent LTP is shown to underlie the antidepressant mechanism of the D1 agonist and D_2/3_ antagonist levo-stepholidine in the medial PFC [107]. The aforementioned findings show distinct rather than directly opposite effects of D_1_- and D_2_-like receptors and indicate that PFC dopamine is critically involved not only in the maintenance but also the gating, induction and expression of long-term synaptic plasticity and support key therapeutic potential in attention defects and psychiatric disorder-related mood and cognitive impairments. 

The second main cortical region receiving VTA dopaminergic inputs is the M1-cortex in which dopamine is found essential in motor skill acquisition and associated long-term plasticity through D1 and D2 receptors [108]. Immunohistochemistry staining-based retrograde tracing provides direct evidence that VTA projections release dopamine in the M1-cortex that subsequently mediates gene expression and plasticity-based motor skill learning but not retrieval, which is mimicked through direct VTA stimulation and blocked by VTA lesioning that is rescued via direct levodopa injection [109]. Similar to the aforementioned results in the PFC, dopamine-mediated LTP is not necessarily PKA-dependent as LTP and motor learning are found to depend on D1 and D2 receptor modulation of phospholipase C (PLC) activation rather than PKA in the M1-cortex [110]. Dopamine also has direct modulatory effects on M1-cortex network activity, neuronal excitability, gene expression, synaptic transmission and LTP induction supporting its roles in the encoding, mapping and storage of skill memory [111]. It is found that D2 but not D1 receptor blockade reduces forelimb representation and elevates movement thresholds. In addition, dopamine denervation results in the loss of LTP, neuronal recruitment and synaptic reorganisations that are required for accurate and refined movement during motor learning [112,113].

#### 4.1.2. Nigrostriatal Pathway

SNc dopaminergic projections extend to the dorsal striatum (DS)—comprising the putamen and caudate nucleus—which receives excitatory glutamatergic inputs from the thalamus and cortex (i.e., corticostriatal pathway) and is suggested to be critically involved not only in movement, as a critical component of the basal ganglia system, but various other functions including cognitive, emotion and motivation-based decision making and learning [114]. The vast majority (95%) of striatal neurons are GABAergic principal medium spiny neurons (MSNs) while the rest are cholinergic interneurons (0.5%–1%) and distinct subtypes of GABAergic aspiny interneurons (3%–4%) [115]. A considerable body of evidence shows key roles for SNc in learning and memory in addition to associated dopamine release in the control of LTP and LTD of striatal MSNs. Early investigations showed that direct stimulation of SNc during learning impairs memory retention, followed by studies directly implicating SNc in spatial/relational learning independently from hippocampal function in rodents and a potential role in reinforcement learning in humans [116,117,118,119]. 

Multiple experimental models of Parkinson’s disease (PD) have been developed, of which the most commonly studied models are based on direct lesioning or selective-toxicity induction of dopaminergic SNc neurons [120,121]. In PD models and patients, the disruption or loss of SNc dopamine is found to impair long-term plasticity in both M1-cortex and DS in which plasticity alterations are believed to mediate PD and L-DOPA associated motor impairments and dyskinesia, respectively [122,123]. The corticostriatal plasticity impairments are found to depend on the level of denervation in which minimal dopamine reductions which correspond to mild/early PD impair NMDAR-dependent LTP but not LTD in contrast to complete depletion, which impairs both [124]. Consistent with this, dopaminergic neuron transplantation in PD rat-model shows that improved motor symptoms are associated with synaptic plasticity restorations that are seen only within the region of the graft [125]. Furthermore, the inability of L-DOPA to rescue M1-cortex plasticity in naïve PD patients despite clinical improvements, while restoring M1-cortex LTP in non-dyskinetic but not dyskinetic patients in some studies in addition to direct evidence in mouse models associating unidirectional striatal plasticity of the direct and indirect pathways in PD symptoms and L-DOPA-induced dyskinesia provide clear evidence for the critical roles of corticostriatal plasticity in motor control and that pathway-selective effects of dopamine mediate plasticity-associated functional alterations under healthy/physiological conditions [126,127,128]. 

Various studies investigating the mechanisms underlying long-term plasticity at corticostriatal synapses show that LTP is readily induced via TBS and HFS, NMDAR-mediated and dependent on D1 receptor activation [129,130]. Furthermore, corticostriatal D1 activation is found essential for the induction of both LTP through a PKA-dependent mechanism and LTD through a PKG-dependent mechanism that are induced in functionally-distinct neuronal populations [131]. On the other hand, t-LTP induced via pairing stimulation requires endocannabinoids (eCB) and presynaptic D_2_-like receptors in contrast to t-LTD that requires D_1_-like receptors and eCB signalling at corticostriatal but not thalamostriatal synapses and is mostly expressed postsynaptically [132,133,134]. 

Despite the lack of structurally-defined subsets of corticostriatal MSNs, two main movement-control and possibly action-selection pathways arising from DS projections have been identified and are classified based on the connection with the basal ganglia “interface” into direct and indirect pathways, both of which being critically modulated by SNc dopamine afferents [135]. Direct-pathway MSNs primarily express D_1_-like receptors while D_2_-like receptors are primarily found on indirect-pathway MSNs, both of which are found to undergo LTP and LTD through different mechanisms as discussed, and are described in detail by Cerovic and colleagues in a recent review (2013) in which the authors propose models explaining the induction and expression of long-term plasticity at both pathways [136]. Interestingly, strain variations in the proportion of long and short splice variants of D_2_ receptors result in differential responses to mesostriatal D_1/2_ co-stimulation in relation to c-Fos expression and neurobehavioural plasticity, which is suggested to affect liabilities to certain psychopathologies [137]. 

Lastly, in relation to long-term synaptic plasticity, dopamine is found to control glutamatergic transmission in the DS through differential D1 and D2 receptor-mediated modulation of intrinsic neuronal excitability via multiple mechanisms through regulating K^+^, Na^+^, VGCC, AMPAR and NMDAR channel currents during transmission that adjust dendritic excitability and subsequently plasticity induction in addition to regulating AMPAR surface expression and presynaptic neurotransmitter release [138,139].

#### 4.1.3. Mesolimbic Pathway

The second main VTA dopaminergic projection innervates the ventral striatum, particularly the nucleus accumbens (NAcc)—comprised of core and shell subregions involved in limbic and motor control—which has long been heavily implicated in various behaviours and cognitive functions such as reward, aversive learning, motivation, addiction, declarative learning and memory [140,141]. The NAcc receives glutamatergic excitatory inputs from five main brain structures or pathways: the PFC, hippocampus, amygdala, thalamus and VTA all of which are modulated by dopamine and proposed to mediate distinctive functions [142,143,144,145]. However, the functional differences between input pathways are not clear and show various interrelated roles; for instance, thalamic–NAcc inputs are found to mediate aversive memory and opiate withdrawal effects following chronic exposure-induced LTP, while amygdala but not PFC inputs facilitate reward seeking. On the other hand, hippocampal–NAcc inputs are found essential for spatial/reference memory and conditioned place preference LTP that is impaired following stress. In addition, PFC–NAcc inputs are potentiated following cocaine self-administration and believed to mediate seeking and withdrawal while VTA glutamatergic inputs on NAcc GABAergic interneurons are shown to drive aversion [146,147,148,149,150,151]. 

NAcc dopamine efflux is found critical in regulating activity-based selection of multiple inputs that either converge monosynaptically, as hippocampal and PFC inputs, or heterosynaptically; for instance, stimulating the hippocampal pathway causes D1-mediated potentiation and inhibits amygdala inputs through D1 and adenosine A_1_ receptors, whereas simultaneous pathway stimulation potentiates both [143,152]. In addition, it was found that direct amphetamine injection, during simultaneous place and cue conditioning, in the NAcc shell and core regions facilitate and impair hippocampal-dependent place conditioning respectively; similarly, blocking dopamine receptors in NAcc shell attenuated place conditioning in contrast to NAcc core dopamine blockade which facilitated and also impaired place and cue-conditioning, respectively [153]. Lastly, one plausible input-selection mechanism is based on lateral inhibition through GABAergic MSN transmission which is found to be attenuated by D1 receptor activation that is suggested to: eliminate competitive pathway interactions, cause a shift in input selection and amplify associated afferent signals [154]. Therefore, NAcc dopaminergic modulation of input signal gains could determine which pathways undergo plasticity changes based on behaviourally relevant stimuli.

The roles of NAcc dopamine in learning and behaviour are well-documented: for instance, in spatial memory consolidation through both D1 and D2 receptors, food-reinforced instrumental learning requiring coincident D1 and NMDAR activation, fear conditioning, reward learning through distinct but still cooperative effects of D1/D2 receptors and motivation, which is suggested to depend on neuronal-calcium sensor 1-mediated regulation of D2 receptors [155,156,157,158,159]. The neuronal composition of the NAcc is very similar to the DS consisting primarily of D_1_- and D_2_-like receptor-expressing MSNs and, in addition to the aforementioned modulatory roles of dopamine, various studies report its direct roles in plasticity induction, particularly LTP at NAcc synapses. 

The D1-mediated activation of PKA in NAcc neurons is found to induce the expression, phosphorylation and synaptic insertion of AMPARs in NAcc cultures; in addition to priming LTP, providing a narrow time-detection mechanism for potentiation and mediating both the induction and maintenance of aversive memory at D2-expressing MSNs [160,161,162,163,164,165]. Furthermore, the induction of LTP in the NAcc requires D1/mGluR5 coactivation in a concentration-dependent manner as elevated dopamine and/or glutamate levels are found to impair associated HFS-induced LTP [166]. On the other hand, little is known about the mechanisms by which dopamine regulates NAcc LTD. Nonetheless, plasticity-related functional and structural alterations in motivational drive and drug abuse provide key insights on NAcc dopamine signalling and possible therapeutic potential in the management of drug addiction and withdrawal [167,168].

#### 4.1.4. Hippocampal Dopamine

The hippocampus has a central role in the study of learning and memory mechanisms with regards to synaptic plasticity and has been the focus of extensive research for many decades with multiple pathway-specific and network-based models [169]. The roles of various neuromodulators were studied in relation to hippocampal plasticity including dopamine. The VTA has been previously considered as the main and only source of hippocampal dopaminergic transmission; however, dopamine is shown to be released from the axons of locus coeruleus adrenergic neurons as well, which are more-densely found in the dorsal hippocampus, particularly the dorsal CA1 and CA3 regions, in contrast to VTA projections that innervate ventral regions with only sparse dorsal presence [170]. The roles of dopamine in relation to hippocampal-dependent learning and memory are well-documented and dopamine is argued to promote more-motivational memories in relation to adaptive behaviour and hippocampal function [171]. 

Interestingly, it was previously proposed that dorsal and ventral regions of the hippocampus mediate distinctive memory subtypes (cognition and emotion-based memories respectively) [172]. More recent evidence suggests that dopaminergic modulation is source/region-dependent and that VTA–ventral and locus coeruleus–dorsal hippocampal inputs are proposed to control two types of novelty-related memories described as “common” and “distinct” novelties, respectively, with regards to the presence or lack of past experience [173]. Similarly, novel-environment exposure results in a brief period of reduced LTP induction threshold through a D1 receptor-mediated facilitation mechanism, suggesting a key role in the retention of unexpected information [174]. Environmental novelty is found to trigger locus coeruleus neuronal firing that subsequently mediates the enhancement of acquired novelty-related memories through dopaminergic D1 but not adrenergic receptors [175]. Dopamine is also found essential for functional coupling of network activities during learning and memory formation; for instance, between the hippocampus and amygdala during the acquisition of fear conditioning through D1 receptors and between the hippocampus and caudate nucleus for episodic memory, which shows positive interrelation with D2 receptor availability in both regions [176,177]. 

In regards to the mechanisms by which dopamine modulates hippocampal long-term plasticity, most studies have focused on the CA1 region. In hippocampal neuronal cultures D1 receptor activation induces dendritic local protein synthesis, GluA1-subunit upregulation, AMPAR surface insertion and enhanced synaptic transmission [178]. Early evidence from acute hippocampal slices showed that D1 receptor activation during LTP induction is required for the late phase of CA1 LTP induced following stratum radiatum (i.e., Schaffer collaterals) tetanisation, as D1 antagonists applied during but not directly following induction impair the late phase, confirmed by a later study showing that D1 mediates the maintenance of HFS-induced Schaffer collateral–CA1 NMDAR-dependent LTP which requires CA1 protein synthesis and that direct D1 activation by itself can induce a slow-onset LTP [179,180]. Interestingly, it was found in a later study that the D1 agonist-mediated LTP requires NMDAR activation as well [181]. Accordingly, D1 receptor activation is found to partly contribute to the magnitude (20%–25%) of early (up to 40 minutes) LTP, an effect mimicked by the adenylyl-cyclase activator forskolin, suggesting a PKA-mediated mechanism [182]. However, a more recent study investigating the slow-onset D1-induced LTP, in CA1 neurons, shows differential dose-dependent roles of MEK (MAPK/ERK pathway) and CAMKII enzymes as both were essential for LTP induction following weak/low-dose D1 activation, whereas with stronger dopaminergic D1 activation the role of CAMKII becomes partial and no longer a prerequisite for LTP induction in contrast to MEK1/2 role which remains necessary [183]. 

CA1 dopamine is found in vivo to facilitate not only late-phase LTP but also late LTD associated with object exploration through D1 receptors; similarly, D2 receptors are essential for the expression of both LTP and LTD as well, the latter of which is found necessary for spatial learning and dependent on presynaptic D2 receptors [184,185]. Interestingly, the D1 agonist SKF83959, which is reported to activate PLC, is found to induce and facilitate LFS-induced LTD, which was dependent on NMDAR, PLC, calcineurin and free cytosolic Ca^2+^, as the LTD facilitation was attenuated using a cell permeable Ca^2+^ chelator (BAPTA-AM) [186]. 

Lastly, subtype-selective effects of D2 receptors in plasticity are also reported; for instance, D_3_ activation significantly increases LTP while D_4_ receptor activation results in complete depotentiation (i.e., LTP reversal) in CA1 neurons and mediates the plasticity effects of neuregulin-1, which has been linked to schizophrenia [187,188].

### 4.2. Cholinergic Transmission in the Hippocampus

The critical involvement of acetylcholine in learning and memory, particularly in relation to acquiring novel information, are well-documented through studying the effects of lesioning and pharmacological antagonism that show region and task-specific functions which are suggested to rely on acetylcholine-mediated afferent input enhancement, spiking persistence and modulation of inhibition [189]. Acetylcholine exerts its actions through two main classes of receptors: ionotropic nicotinic (nAchR) and metabotropic muscarinic (mAchR) acetylcholine receptors that include various subclasses. Nicotinic receptors are ligand-gated cation channels the permeability of which relies on the subunit composition; generally, most subtypes are permeable to Na^+^ and K^+^ while α7-nAchR shows rapid sensitisation and significant Ca^2+^ permeability [190]. In relation to synaptic plasticity, most evidence exists for α7-nAchR, which has been shown to be heavily involved in Alzheimer’s disease pathology in addition to cognition and memory processes [191,192]. On the other hand, five muscarinic receptor subtypes have been identified and are either coupled to Gq (M_1_, M_3_ and M_5_ subtypes) or Gi (M_2_ and M_4_ subtypes) G-proteins and show differential distribution across multiple brain regions and synaptic loci of expression [193]. 

Three main populations or nuclei of cholinergic neurons have been identified; the medial septal nucleus, which is the main source of hippocampal acetylcholine, the basal nucleus of Meynert (NBM) which mostly innervates the cerebral cortex and the pedunculopontine tegmental nucleus, all of which are suggested to coordinate cognitive processes [194,195]. Moreover, cholinergic (i.e., acetylcholine releasing) interneurons are known contributors to synaptic plasticity in specific synaptic populations, particularly at corticostriatal synapses [196]. With regards to long-term synaptic plasticity, cholinergic pathways have not been characterised as well as dopamine; however, the most studied cholinergic system is the septohippocampal pathway—encompassing the hippocampal formation, the septum and their interconnections in addition to afferent and efferent pathways—which has been heavily implicated in memory-related processes including information encoding, retrieval and consolidation [197]. As previously mentioned, neuromodulators induce synchronous neuronal activity associated with synaptic plasticity as well as memory and of particular importance in relation to the hippocampus is the septal cholinergic transmission which is often described as the theta-rhythm generator, which is required for hippocampal LTP [66,198]. Moreover, evidence suggests intrinsic theta-generating capabilities of the hippocampus that, together with septum-originating rhythms, rely heavily on muscarinic receptors [199,200]. 

Muscarinic receptors are essential for various types of learning and memory functions; for instance, M_1_ receptor blockade impairs emotional and spatial memory as well as the retrieval of fear conditioning while blocking presynaptic M_2_ autoreceptor enhances acetylcholine release and subsequently improves spatial memory and cognitive performance in contrast to M_2_ knockout mice that exhibit impairments in working memory and acquisition but not reference memory or retention [201,202,203,204,205,206]. Evidence shows key memory roles for hippocampal M_3_ and M_4_ receptors, the latter of which is significantly reduced in Alzheimer’s disease and its activation, and, in addition to M_1_ receptor, is proposed as a novel treatment approach in Alzheimer’s disease [207,208,209,210]. Conversely, the molecular mechanisms by which muscarinic receptors modulate synaptic plasticity and cognitive functions are less understood; however, evidence shows critical modulatory roles on hippocampal network functions accomplished via coordinated muscarinic and nicotinic receptor activation [211]. Briefly, presynaptic muscarinic receptors (i.e., M_2_ and M_4_) regulate neurotransmitter release while postsynaptically expressed (i.e., somatic and dendritic) in addition to astrocytic and inhibitory interneuron receptors control their excitability, through multiple mechanisms, via precise spatiotemporal regulation arising from both phasic and tonic release which is supported by evidence showing that synergistic activation of multiple muscarinic receptor subtypes is essential for both memory acquisition and retrieval [212,213]. 

Various studies have directly investigated the roles of muscarinic receptors, particularly M_1_, in the induction and maintenance of long-term synaptic plasticity in the hippocampus and show timing and concentration-dependent effects. Early evidence shows that HFS-induced LTP at CA3–CA1 synapses can be enhanced by the activation of M_1_ receptors using low carbachol concentration or direct stimulation of acetylcholine release, and both effects were abolished in M_1_ but not M_3_ knockout mice despite that LTP was preserved. In contrast, high concentrations of carbachol resulted in short-lasting synaptic inhibition, which was impaired in M_1_ and M_1_/M_3_ knockout mice [214]. These results were confirmed in a later study in which M_1_ activation facilitated TBS-induced LTP through inhibiting postsynaptic small conductance Ca^2+^-activated K^+^ (SK) channels, thereby preventing their hyperpolarising effect leading to enhanced NMDAR channel opening/activation during TBS and subsequently LTP facilitation [215]. Moreover, direct agonist activation of M_1_ receptors significantly increases neuronal excitability, enhances spike-coupling and is found sufficient to induce robust NMDAR-dependent LTP that occludes, and therefore shares similar mechanisms with, stimulus/TBS-induced LTP at CA3–CA1 synapses [216]. Interestingly, the activation of not only surface but also intracellular M_1_ receptors enhances LTP, the latter of which is mediated through the phosphorylation of ERK1/2 [217]. It was found in vivo as well that the muscarinic M_1_ receptor is required for CA1 LTP induction and that acetylcholine preconditioning through stimulating septal release provides a narrow time-window of lowered LTP induction threshold that was prevented when atropine was applied [218]. Lastly, a special form of anti-Hebbian LTP induced at inhibitory CA1 interneurons that favour postsynaptic hyperpolarisation is found dependent on the co-activation of M_1_ and group I mGluR, which shows a new mechanism by which cholinergic inputs regulate network transmission as this form of potentiation results in a long-term increase of inhibition that is suggested to regulate input/pathway selection [219]. 

The M_1_ receptor is also involved in LTD induction; for instance, presynaptic (CA3) M_1_ receptors are found essential, through a PKC-dependent mechanism, for the induction of mGluR cLTD [220]. In addition, LTD induced through direct agonist activation of M_1_ receptors in the hippocampus is shown to rely on different molecular mediators that lead to AMPAR endocytosis compared to mGluR-cLTD despite sharing the same G-protein subtype (i.e., Gq); moreover, another form of M_1_-induced NMDAR-dependent LTD is found dependent on IP_3_-mediated intracellular Ca^2+^ release and is expressed via NMDAR internalisation [221,222]. On the other hand, the molecular mechanisms by which M_2–5_ muscarinic receptors modulate synaptic plasticity are less understood despite being critically involved in learning and memory as previously discussed; however, the different subtypes of muscarinic receptors exhibit different roles in plasticity modulation [223]. Ultimately, the substantial involvement of acetylcholine in cognitive processes and synaptic plasticity in addition to network function modulation, neuronal synchronisation and neurodegenerative disorders, particularly Alzheimer’s disease, warrant further investigation.

### 4.3. Adrenergic Transmission

Noradrenaline (norepinephrine) in the brain has fundamental roles in various behaviours, namely: arousal, attention, motivation, stress and vigilance. In addition, emerging evidence supports its key roles in learning and memory [224]. Noradrenaline is exclusively released from a specific group of brainstem nuclei (A1–A7), predominantly locus coeruleus, that project and supply noradrenaline across various brain regions and of particular importance in relation to cognitive function are the PFC and the hippocampus [225]. Noradrenaline targets two main classes of receptors, namely α and β adrenoceptors. The former comprises α_1_ and α_2_ receptors, which are coupled to Gq and Gi proteins, respectively, while the latter comprises β_1_, β_2_ and β_3_ receptors, all of which are coupled to Gs protein while β_2/3_ receptors are also coupled to Gi. 

In relation to learning and memory functions, noradrenaline release in the hippocampus, within a narrow time window of learning/acquisition, is found essential in memory formation and consolidation. In addition, the key modulatory roles of noradrenaline in emotional/stress, fear and spatial working memories are well-documented [226,227,228,229,230]. Accordingly, noradrenaline release from the locus coeruleus is proposed to control cognitive flexibility and orientation of attention as supported by evidence showing spatiotemporal association between noradrenaline release and different brain states through both brain-wide and region-specific activity modulation, especially in relation to hippocampal and cortical oscillations [231,232]. 

It has been recently found that dopamine is also released from locus coeruleus adrenergic neurons [170], which would require reinterpretation of earlier findings that investigated noradrenaline role in synaptic plasticity through locus coeruleus stimulation, or activity monitoring, without pharmacological confirmation of adrenoceptor involvement. Therefore, the molecular mechanisms by which noradrenaline regulates synaptic plasticity are reviewed based on adrenoceptor-selective effects. The strongest evidence in relation to synaptic plasticity exists for β_2_ receptors; however, other adrenoceptors have been shown to not only be involved in synaptic plasticity and memory but also exhibit complex interactions requiring the combined effects of multiple receptor subtypes as will be discussed. 

Early evidence showed that α_1_ receptor activation facilitates spatial learning despite not being an essential requirement for acquisition [233]. This was later confirmed using mutant mice expressing a continuously active form of α_1A_ receptor subtype. These exhibited enhanced hippocampal LTP induction and cognitive performance, in contrast to α_1A_ knockout mice which exhibited poor cognitive functions [234]. Interestingly, the simultaneous blockade of α_1_- and β-type adrenoceptors is found to impair spatial avoidance learning; however, the antagonism of either receptor separately was unable to impair locomotion, which suggests an adrenoceptor interaction required for the regulation of navigation and learning [235]. Similarly, α_1A_ and β_1_ adrenoceptors provide bidirectional regulation of the magnitude of kainate-induced hippocampal gamma oscillations, which illustrates how noradrenaline can either promote or depress oscillations with possible relation to information processing in learning and memory [236]. In contrast, antagonising α_1_ receptors is found to facilitate LTP and fear conditioning in the amygdala through suppressing and promoting inhibitory and NMDAR currents, respectively. In addition, α_1_ antagonism is also shown to dose-dependently potentiate NMDA but not AMPA or kainate receptor currents in hippocampal CA1 neurons resulting in significant enhancement of spatial learning and memory [237,238]. These results show how noradrenaline, through α_1_ receptors, bidirectionally regulates synaptic plasticity and memory formation not only through regulating LTP induction/maintenance but also through direct LTD induction, as previously reported in the hippocampus that α_1_ receptor activation induces NMDAR-dependent LTD, which requires pre- and postsynaptic neuronal activity and is independent on the activation of GABA_A_ receptor or other adrenoceptors [239]. 

α_2_ adrenoceptors mostly serve an inhibitory presynaptic autoreceptor role and their in vivo activation is reported to impair HFS-LTP induced at Schaffer collaterals CA3–CA1 synapses in a dose-dependent manner that was accompanied with decreased hippocampal overall neuronal glutamate content and increased paired-pulse facilitation ratio (i.e., reduced presynaptic glutamate release), which is suggested to be mediated through inhibiting HCN channels (i.e., hyperpolarisation-activated cyclic nucleotide-regulated channels) [240]. Interestingly, the activation of α_2_ receptors in the basolateral amygdala was shown in vivo to augment HFS-induced LTP at hippocampal–PFC synapses while its blockade impaired LTP induction; as opposed to the non-selective activation and blockade of β receptors in the amygdala which reduced and enhanced LTP respectively at the same synapses [241]. 

β adrenoceptors, particularly the β_2_ subtype, have been more-heavily investigated in relation to synaptic plasticity induction and maintenance. In the hippocampus, a recent study shows that β adrenoceptors selectively augment LTP in the ventral CA1 region, which shows higher adrenergic sensitivity compared to the dorsal region, leading to enhanced TBS-induced LTP magnitude and stability. In addition, the exogenous application of isoprenaline resulted in increased postsynaptic excitability, facilitated VGCC-mediated LTP and the induction of robust NMDAR-dependent LTP following subthreshold primed stimulation in the ventral CA1 in contrast to moderate burst response enhancement in dorsal CA1 region [242]. This is in accordance with more recent findings that the source of hippocampal dopamine is region-dependent as ventral regions are mostly VTA innervated while dorsal regions receive dopamine from locus coeruleus [173], suggesting distinct modulatory roles between hippocampal dopamine and noradrenaline. Additionally, the facilitated induction of hippocampal prefrontal path LTP, following weak electrical stimulation, through the application of methylphenidate which inhibits the reuptake of dopamine and noradrenaline is found to rely on both β and, to a larger extent, D1 receptors leading to improved working memory [243]. Moreover, the combined activation of β_1_ receptor and M_1_ muscarinic receptor using isoprenaline and carbachol, respectively, shows synergistic activation of mTOR, ERK and protein translation in hippocampal CA1 neurons leading to the transformation of stimulus-induced short-term potentiation into sustained LTP [244]. Despite some studies using exogenous agonist application, which may be argued to be an imperfect physiological representation, these results show novel forms of interactions hinting towards intracellular coincident detectors downstream of GPCR activation. 

Multiple studies selectively investigated β_2_ adrenoceptors which have been shown to play critical plasticity roles through various mechanisms; for instance, β_2_ activation and subsequent cAMP-PKA pathway stimulation enhance the magnitude of LTP at hippocampal CA3–CA1 synapses through AMPAR GluA1-subunit phosphorylation and augment t-LTP at PFC Layers II/III lateral synapses through postsynaptic PKA activation and suppression of GABA_A_-mediated inhibitory transmission [245,246]. Additionally, the kinase-anchoring protein gravin has been identified—using knockout mice lacking its α-isoform which exhibit plasticity and selective memory impairments—as an essential mediator for PKA-induced β_2_ receptor phosphorylation and memory formation-associated ERK activation in the hippocampus. This suggests that gravin protein provides a molecular coupling mechanism and shows further interactions between glutamatergic and adrenergic transmission [247]. Similarly, a recent study investigating synergistic cAMP production in response to simultaneous β/NMDA receptor activation, using isoprenaline and NMDA respectively as an LTP mechanism, showed that, when β adrenoceptor activation precedes NMDARs (by several minutes), it results in the attenuation of the NMDAR–cAMP response that through simulation modelling suggests a novel “Gs-to-Gi” adrenoceptor switch in response to PKA-mediated β_2_ receptor phosphorylation and subsequent Gi-mediated adenylyl cyclase inhibition [248]. Lastly, other β_2_-mediated plasticity mechanisms have been identified and include the phosphorylative enhancement of L-type VGCC and subsequent LTP induction in addition to the inactivation and removal of dendritic Kv1.1 channels resulting in increased excitability, depolarisation, coincident activity detection and subsequently the facilitation of t-LTP at hippocampal CA3–CA1 synapses [249,250].

### 4.4. Serotonergic Transmission

Serotonin (5-hydroxy tryptamine, 5-HT) has essential roles in the control of a wide-range of functions including sensory and motor modulation, emotion regulation and cognitive control in addition to its critical involvement in the pathophysiology of various disorders such as migraine, anxiety and depression [251]. The exclusive source of brain serotonin is a group of nuclei (B1–B8) collectively referred to as the Raphe nuclei which project and release serotonin throughout the brain. Serotonin targets seven main receptor types (i.e., 5-HT_1–7_) that are further subdivided into at least 14 different subtypes, all of which are GPCRs except for 5-HT_3_ which is an ionotropic receptor permeable to Na^+^, K^+^ and Ca^2+^ ions. Metabotropic serotonin receptors are coupled to different G-proteins including Gi (5-HT_1_ and 5-HT_5_), Gq (5-HT_2_) and Gs (5-HT_4_, 5-HT_6_ and 5-HT_7_) [252]. 

The crucial roles of serotonin receptors in learning and memory are well-established; for instance, 5-HT_1A_ is necessary for object recognition memory while 5-HT_2C_ is required for stress-induced consolidation of fear memory [253,254]. On the other hand, the chronic activation of 5-HT_4_ is found to reverse episodic and spatial memory impairments in mouse models of anxiety and depression in addition to passive avoidance and spatial memory in scopolamine-treated mice [255,256]. In contrast, the blockade of 5-HT_6_ receptor enhances object recognition memory, whereas 5-HT_7_ activation improves spatial, cognitive and contextual memory with suggested therapeutic potential in autism and fragile-X syndrome [257,258]. 

However, serotonin remains the least characterised neuromodulator in relation to its molecular mechanisms of plasticity regulation as most studies do not undertake a receptor subtype-selective approach. Nonetheless, a few studies investigated the roles of 5-HT_2_ receptor and show similar mechanisms to other neuromodulators, in the regulation of glutamatergic transmission and subsequent plasticity induction in various brain regions. In the basolateral amygdala, 5-HT_2_ receptor activation transforms TBS-induced short-term potentiation into LTP through enhanced NMDAR-mediated potentials and calcium influx; consistently, the presynaptic 5-HT_2A_ receptor subtype in the PFC is also shown to enhance NMDAR transmission, thereby gatekeeping subsequent induction of presynaptic t-LTD at corresponding thalamocortical synapses [259,260]. Similarly, the application of LFS in the NAcc stimulates serotonin release and LTD induction through 5-HT_2_ receptor-mediated enhancement of L-type VGCC influx and eCB release [261], while in the PFC serotonin facilitates tetanic stimulation-induced NMDAR-independent LTD through 5-HT_2A_ and group-I mGluR synergistic p38-MAPK activation and subsequent AMPAR internalisation [262]. 

Lastly, serotonin is able to modulate glutamate and GABA-mediated excitatory and inhibitory transmission, respectively, through various receptor subtype-selective mechanisms such as AMPAR recruitment, NMDAR conductance modulation, cooperative GABA_B_ interaction, GABA_A_ phosphorylation and neurotransmitter release regulation [263].

## 5. Astrocytes and Neuromodulators

Astrocytes are increasingly recognised as active signalling elements that exhibit the abilities to adjust neuronal excitability and synchronous activity [264]. In addition, astrocytes are heavily implicated in synaptic plasticity and are found to undergo plasticity changes in response to neuronal activity [265]. Astrocytic perisynaptic processes express a wide range of receptors the activation of which triggers astrocyte excitation—through the generation of dynamic intracellular Ca^2+^ transients that rely primarily on the Gq–IP_3_ pathway (IP_3_R_2_ in astrocytes)—and induces subsequent release of gliotransmitters; thus, providing a bidirectional communication mechanism [266,267]. Moreover, astrocytes express the receptors of, and respond to a vast number of neuromodulators [268]; hence, astrocytes are able to detect and respond to both neuromodulatory signals and neuronal activity.

**Figure 2:**
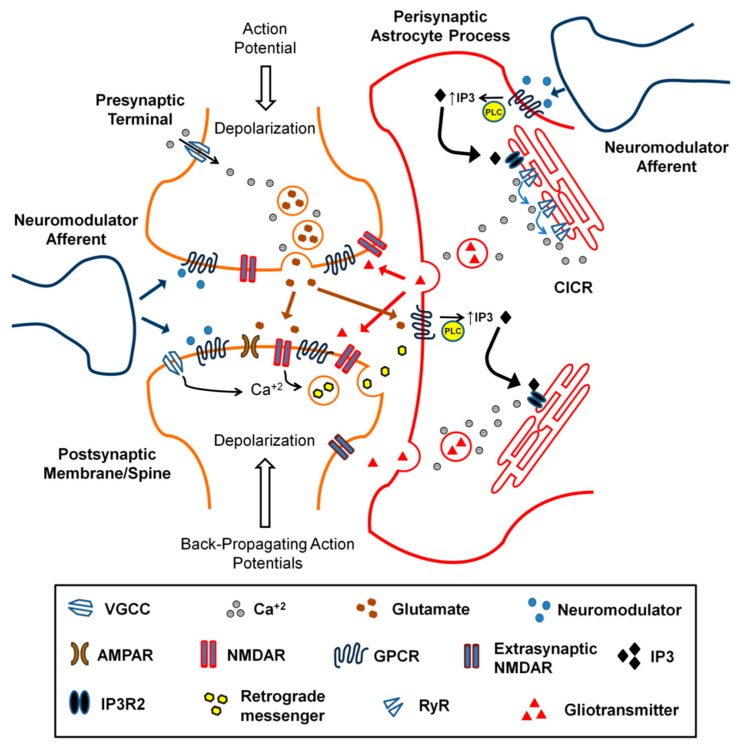
A schematic representation of astrocytic synaptic coverage and neuromodulatory inputs. Astrocytes are able to detect and respond to both synaptic activity and neuromodulatory afferent signals. Neuromodulators and neuron-released transmitters (presynaptic and postsynaptic retrograde messengers) activate associated receptors on perisynaptic astrocyte processes leading to intracellular calcium signalling and subsequent induction of gliotransmitter release to modulate local activity and synaptic plasticity. Similarly, neuromodulatory signals can target neurons, astrocytes or both; hence, neuromodulators transmit behaviour-related signals to induce neuronal activity directly and/or through astrocytes.

Accumulating evidence shows that astrocytes are key mediators of neuromodulator actions. In regards to rhythmic activity and synaptic plasticity, astrocytes are critically involved in the regulation of synchronous neuronal activity and associated processes, possibly in response to neuromodulator signals, as astrocytes are found necessary for cognitive flexibility and theta/gamma coupling in addition to delta-, alpha- and gamma-power oscillations [269]. Accordingly, astrocytes exhibit neuromodulatory interactions in relation to network activity regulation; for instance, astrocytes respond to septal-released acetylcholine through α7-nAchR, via increased glutamate release and subsequent activation of hilar interneurons in the dentate gyrus, hence performing an essential intermediary role of septal cholinergic modulation of hippocampal activity [270]. Moreover, noradrenaline in the visual cortex is found necessary for astrocytic sensitisation to localised neuronal activity which occurs in a behaviour-dependent manner [271]. Additionally, it was found in vivo that somatosensory cortex astrocytes show wide spread Ca^2+^ signalling responses following electrical stimulation of locus coeruleus and peripheral sensory stimulation, through α-adrenoceptors, and this response was found independent on sensory-driven glutamatergic pathways [272]. The above findings indicate a potentially central role for astrocytes in the neuromodulation of network activity, synaptic plasticity and sensory signal processing. A schematic representation of astrocytic synaptic coverage in relation to afferent neuromodulatory signals is shown in Figure 2. Despite being key regulators of neuronal transmission and a major target for neuromodulators, astrocytes have not been adequately studied in relation to neuromodulator roles in synaptic plasticity; nonetheless, recent and emerging evidence shows critical roles for astrocytes in various brain regions. 

In the hippocampus, the activation of muscarinic receptors is found to induce coincident activity between postsynaptic neurons and astrocytes leading to glutamate release that targets mGluRs to induce LTP which can be prevented via: buffering astrocytic calcium, deleting IP_3_R_2_ receptors which mediate astrocytic intracellular calcium release (IP_3_R_2_-kockout mice) or inhibiting G-protein signalling in astrocytes, and mimicked by direct astrocyte stimulation and calcium uncaging when combined with neuronal depolarisation [273]. Furthermore, in the somatosensory cortex, in vivo activation of astrocytic muscarinic receptors following combined NBM and whisker stimulation results in d-serine release and subsequent activation of NMDAR-dependent LTP which was also impaired in IP_3_R_2_-knockout mice [274]. Astrocytic α_1_ adrenoceptor activation induces astrocytic ATP/D-serine release and subsequent LTP induction in the somatosensory cortex [275], while, in the hippocampus, astrocytic but not neuronal β_2_ adrenoceptors are necessary for fear memory consolidation [276]. In addition, deleting astrocytic β_2_ adrenoceptor significantly impaired learning and hippocampal LTP in aged mice despite intact physical health and motor functions in mutant ones [277]. In relation to dopamine, a subset of astrocytes has been shown to express vesicular monoamine transporter-2 (VMAT2), which is necessary for dopamine homeostasis, and its conditional deletion from astrocytes in the developing PFC is found to impair neurotransmission, executive function and LTP [278]. 

A recent finding shows that hippocampal t-LTD (induced via LFS combined with postsynaptic depolarisation) is dependent on astrocytic and not neuronal p38α-MAPK which is responsible for increased gliotransmitter (glutamate) release and astrocyte to neuron communication during LTD induction while its selective deletion leads to long-term memory enhancement in vivo [279]. Interestingly, the requirement of p38-MAPK activation has been observed for NMDAR-dependent hippocampal t-LTD previously [280], which was presumed to be purely neuronal. Similarly, endocannabinoids acting as retrograde messengers have been found essential for t-LTD induction at different synapses, for instance, in the hippocampus (CA3–CA1) and developing somatosensory cortex (Layers IV–II/III) through CB_1_ receptors [281,282]. However, at the same synapses, more recent evidence shows that t-LTD requires endocannabinoid-induced CB_1_-mediated activation of astrocytic Ca^2+^ signalling and subsequent gliotransmitter (d-serine/glutamate) release [283,284]. These findings indicate that astrocytic control of neuromodulatory actions and synaptic plasticity is critical and necessitate further attention.

## 6. Neuromodulators Steer Glutamatergic Transmission and Plasticity

Experimental findings illustrate various mechanisms by which neuromodulators govern the induction and maintenance of long-term synaptic plasticity across multiple brain regions through steering excitatory glutamatergic transmission on multiple scales. First, at the network level, neuromodulators regulate synchronous neuronal activity thereby fulfilling the first previously-identified criterion for plasticity induction which is associativity (i.e., coincident activity between pre- and post-synaptic neurons) [285]. This is based on the generation of single-band or nested network rhythms mostly representing theta-, alpha- or gamma-power oscillations, which are shown to drive overall network plasticity, synaptic plasticity, STDP and episodic memory [286,287,288]. In addition, neuromodulator-regulated oscillations across distinct neuronal populations may provide a mechanism for cross-network coupling. This is analogous to the evident role of modulators in input pathway selection that presumably represents a signal amplification and possibly synapse-selection process. The molecular mechanisms underlying the regulation of synchronous neuronal activity are diverse and mostly rely on the spatiotemporal modulation of intrinsic neuronal excitability and regulation of inhibitory transmission [289]. 

Various neuromodulators have been identified in a considerable number of studies both in vitro and, in particular, in vivo to be essential for the induction of long-term plasticity at specific synapses, which is the second main neuromodulatory function, rather than having a “supplementary” role. This is despite the use of plasticity-inducing stimulation protocols that bypass the requirement of synchronous (i.e., coincident) activity which is one mechanism by which neuromodulators, through rhythmic neuronal activity, control synaptic plasticity induction, and therefore indicating other neuromodulatory mechanisms that are critical for induction.

On a synaptic scale, neuromodulators are found to control the activity, strength and dynamics of glutamatergic synapses via multiple similar and modulator-specific mechanisms such as altering glutamate release and ionotropic (i.e., AMPA and NMDA) receptor conductance. Additionally, neuromodulators regulate glutamatergic responses on the cellular level as well; for instance, though adjusting voltage-gated currents, membrane properties and intracellular messenger systems [290]. Accordingly, the third main role for neuromodulators is to gate the induction and control the polarity of plasticity especially in relation to STDP through regulating the threshold and time-window of induction, respectively [291,292].

The fourth key function of neuromodulators in plasticity is based on the previously mentioned “supplementary” role which in the literature is commonly referred to as a plasticity facilitation mechanism. This can take two main forms: the facilitation of plasticity induction following a “sub-threshold” stimulus, which is comparable to the conversion of short-term into long-term plasticity, which presumably reflects acquisition enhancement, or the augmentation of long-term plasticity, specifically LTP, which mostly represents a slow increase in the magnitude of potentiation. The augmentation of LTP has been observed for various neuromodulators and in the vast majority of cases is PKA mediated and expressed via multiple mechanisms in relation to AMPAR and/or NMDAR function. However, the additional PKA component is argued to serve more intriguing and pivotal roles. The first previously-suggested function of PKA is to provide a synaptic tag that would allow the capture of plasticity gene products required for maintenance [293]. The second possible role is based on epigenetic modifications and gene-expression alterations that would arguably serve a memory stabilisation mechanism required for consolidation as seen with noradrenaline and hippocampal LTP maintenance [294,295]. 

**Figure 3:**
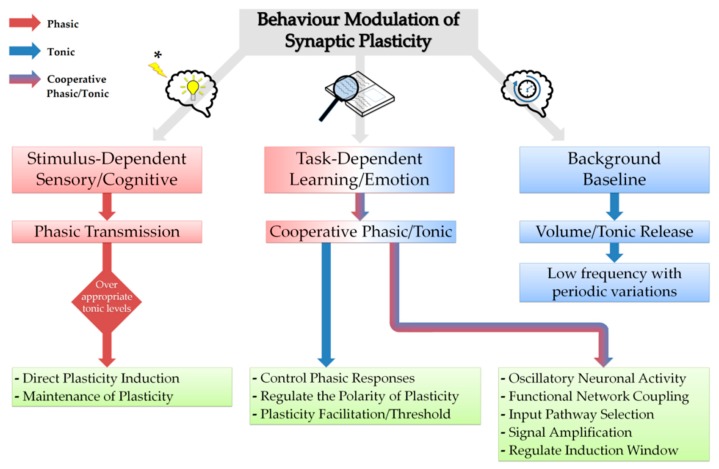
A model of behaviour-dependent neuromodulation of synaptic plasticity. Tonic release of neuromodulators controls baseline and task-induced levels which mediate attention. The cooperative actions of tonic and phasic release during learning tasks modulate network activity and neuronal oscillations that prime plasticity induction through functional coupling, pathway selection and signal amplification. Salient stimuli such as unexpected novelty and reward cues (in rodent models) trigger sub-second phasic transmission to provide the induction/maintenance signal provided that background/tonic levels are “appropriate” for induction. * Salient stimulus.

Another key aspect of particular interest in relation to possible plasticity and memory functions is the spatiotemporal release pattern of neuromodulators—reflecting certain tasks and environmental stimuli such as reward, uncertainty and novelty—that is governed by the timing of release, nature of diffusion, duration of action and receptor distribution in target brain region. It is found that the release of neuromodulators is behaviour-dependent and their cognitive functions require a balance of tonic and phasic transmission that rely on tasks and novel/unexpected stimuli, respectively, and that tonic/baseline levels determine phasic transmission responses as observed for noradrenaline [296,297,298], acetylcholine [299,300,301], serotonin [302,303] and dopamine [304,305,306,307]. Furthermore, tonic levels control phasic actions in an inverted-U-shaped effect in which very low or high background concentrations eliminate phasic-induced responses as observed for dopamine in working memory and cognition [308] in addition to determining the polarity and magnitude of long-term synaptic plasticity [88,307]. Similarly, the inverted-U-shaped effect has been described for acetylcholine, serotonin and noradrenaline as well [309,310,311] and is a shared neuromodulator property involved in attention-induced neuronal activity modulation [312]. A proposed model for neuromodulatory control of plasticity in relation to behaviour-dependent release patterns is shown in Figure 3. 

These findings indicate complex finely-tuned interactions between neuromodulators, especially when considering the intersecting signalling pathways and the relatively slow kinetics of metabotropic regulation when compared to glutamatergic ionotropic transmission, instead of isolated mutually-exclusive functioning [313]. Accordingly, some studies show complex metabotropic neuromodulatory interactions in synaptic plasticity with considerable therapeutic potentials; for instance, amphetamine-induced β adrenoceptor activation rescues D1-mediated LTP in the PFC in dopamine transporter knockout mice, which resembles the hyperdopaminergic state in schizophrenia and attention defective disorder [105]. Additionally, the activation of M_4_ receptor alleviates dyskinetic symptoms and prevents abnormal D1-mediated striatal LTP using an l-DOPA-induced dyskinesia model [314]. On the other hand, antagonising group-I mGluRs alleviates PFC D1-mediated LTP in a mouse model of fragile-X syndrome and improves learning when combined with a D1 agonist [315]. 

Another aspect in relation to neuromodulation of transmission and excitatory/inhibitory balance is the plasticity of synaptic inhibition reported in hippocampal pyramidal neurons. This has been observed particularly for GABA_B_-mediated slow inhibitory currents through G-protein regulated inward rectifying K^+^ (GIRK) channels in vitro [316] as well as in vivo for LTP of GABA_A_ inhibitory postsynaptic responses with potentially key roles in the control of excessive excitation in early Alzheimer’s pathology [317]. Interestingly, GIRK channels are found to be coupled to and regulated by various neuromodulator receptors [318]. Therefore, modulation of synaptic inhibition is another mechanism by which neuromodulators could shape glutamatergic transmission and synaptic plasticity.

The numerous roles of neuromodulators with regard to synaptic plasticity in learning, memory and behavioural functions provide significant insights into the molecular changes underlying certain pathologies, thereby unveiling multiple novel treatment approaches. Alzheimer’s disease represents the most challenging disorder in relation to synaptic plasticity impairments due to its complex underlying pathological alterations. Most neuromodulators have been heavily implicated in Alzheimer’s disease including other newly investigated modulator-related molecules, the roles of which are still emerging such as neurosteroids, cytokines, neuropeptides and peripherally-derived metabolic regulators. For instance, bile acids have recently been shown to indirectly affect synaptic plasticity and improve Alzheimer’s cognitive impairments in rodent models [319,320]. However, the roles of neuromodulators in Alzheimer’s disease-associated plasticity impairments remain unclear. Moreover, since Alzheimer’s disease is characterised by significant imbalance in neurotransmission and modulation, it is important to understand the interaction between deficits in Alzheimer’s disease and how these are affected by current and emerging therapeutic interventions [321,322]. 

Further research is required to explore behaviour-dependent neuromodulatory interactions and how astrocytes are involved in transmitting modulatory signals into synaptic activity and long-term plasticity. Ultimately, neuromodulators are key participants in synaptic plasticity and associated cognitive functions through corresponding GPCRs and their therapeutic potentials extend to cover mood and psychiatric and neurodegenerative diseases.
